# Peer Review of “Mass Testing With Contact Tracing Compared to Test and Trace for the Effective Suppression of COVID-19 in the United Kingdom: Systematic Review”

**DOI:** 10.2196/28745

**Published:** 2021-04-12

**Authors:** Milad Asgari Mehrabadi

**Affiliations:** 1 Department of Electrical Engineering and Computer Science University of California Irvine Irvine, CA United States

**Keywords:** COVID-19, SARS-CoV-2, test and trace, universal testing, mass testing, contact tracing, infection surveillance, prevention and control, review

*This is a peer-review report submitted for the paper “Mass Testing With Contact Tracing Compared to Test and Trace for the Effective Suppression of COVID-19 in the United Kingdom: Systematic Review.”*

## Round 1 Review

### General Comments

This review paper [1] assessed the importance of mass testing in controlling the spread of COVID-19 in the United Kingdom.

### Specific Comments

This is a great, comprehensive work; congratulations to the author. However, I have some suggestions:

1. The information provided in the *Methods* section is extra and should not be mentioned there. The *Methods* section covers the methodology of the study, not the background.

2. The references to the studies mentioned in the *Results* section do not follow the journal’s requirements. Please kindly fix those.

3. For Table 1, my suggestion is to add more columns explaining the summary of the study in a structured format. For example, you can distribute the information you have in the description as number of participants, the country under study, etc.

4. Do not repeat the methodology in the *Results* section.

5. Headers in the *Results* section do not follow the journal’s requirements.

6. Put abbreviations used in the paper at the end of the paper as well (follow journal style).
